# An update on CAD-CAM usage for removable partial denture fabrication: A systematic review

**DOI:** 10.6026/9732063002001794

**Published:** 2024-12-31

**Authors:** Rajeev Singh, Gaurang Mistry, Manju Choudhary, Sheetal Parab, Rasha Ansari, Sanpreet Singh Sachdev

**Affiliations:** 1 Department of Prosthodontics, D.Y. Patil Deemed to be University, School of Dentistry, Navi Mumbai, Maharashtra, India; 2 Department of Oral Pathology and Microbiology, Bharati Vidyapeeth (Deemed to be University) Dental College and Hospital, Navi Mumbai, Maharashtra, India

**Keywords:** Computer-Aided Design, Computer-Aided Manufacturing, removable partial dentures, prosthodontics, framework

## Abstract

The accuracy of Computer-Aided Design and Computer-Aided Manufacturing (CAD-CAM) systems in the fabrication of removable partial denture (RPD) frameworks compared to conventional manufacturing methods is of interest to dentists. Known data show that CAD-CAM systems produce RPD frameworks with superior fit and adaptation, potentially reducing post-insertion adjustments and enhancing patient satisfaction. The importance of digital impressions, advanced CAD software and the capabilities of milling or 3D printing equipment in determining the success of CAD-CAM fabricated frameworks is highlighted. Despite promising results, further research is needed to evaluate the long-term clinical performance of CAD-CAM systems in RPD fabrication and to address the existing limitations.

## Background:

The advent of Computer-Aided Design and Computer-Aided Manufacturing (CAD-CAM) technology has revolutionized the field of prosthodontics, particularly in the fabrication of removable partial dentures (RPDs) [1]. Traditionally, RPD frameworks have been manufactured using conventional techniques such as casting, which, despite being well-established, are associated with several limitations, including material shrinkage, inaccuracies in fit and time-consuming processes [2]. The integration of CAD-CAM systems offers a potential solution to these challenges by providing a more precise, efficient and reproducible method for fabricating RPD frameworks [3]. CAD-CAM technology enables the digital design and automated milling or 3D printing of prosthetic frameworks, leading to improvements in fit, strength and overall quality. The accuracy of the RPD framework is critical, as it directly influences the fit of the denture, patient comfort and the long-term success of the prosthesis [4]. Several studies have indicated that CAD-CAM fabricated frameworks demonstrate superior fit and adaptation compared to those produced by conventional methods, potentially reducing the need for post-insertion adjustments and enhancing patient satisfaction [5, 6-7]. The accuracy of CAD-CAM systems in RPD framework fabrication depends on several factors, including the precision of the digital impression, the quality of the design software and the capabilities of the milling or printing equipment [1]. Digital impressions captured using intraoral scanners or extraoral scanning systems are highly accurate, thereby contributing to the overall precision of the CAD-CAM process [8]. Furthermore, advancements in CAD software have enabled more sophisticated designs that optimize the distribution of forces and enhance the biomechanical properties of the prosthesis [9]. Despite the promising advantages of CAD-CAM technology, some challenges and limitations need to be addressed [10]. The high initial cost of equipment, the learning curve associated with mastering the technology and the need for skilled technicians to operate the systems are some of the barriers to widespread adoption. Additionally, the accuracy of the final product can be influenced by various factors throughout the digital workflow, including the type of materials used, the resolution of the scanner and the parameters set during the milling or printing process [11]. Therefore, it is of interest to provide a comprehensive analysis of the current evidence on the accuracy of CAD-CAM systems in fabricating RPD frameworks, comparing them with traditional manufacturing techniques.

## Methods and Materials:

## Search strategy:

An extensive literature search was conducted to identify studies assessing the accuracy of CAD-CAM systems in the fabrication of removable partial denture (RPD) frameworks. The databases searched included PubMed, Scopus, Web of Science and Cochrane Library. The search spanned from the inception of these databases from January 2023 to September 2024. A combination of keywords and Medical Subject Headings (MeSH) terms such as "CAD-CAM systems," "removable partial dentures," "accuracy," "framework fabrication," and "digital dentistry" were used. Additionally, the reference lists of the selected articles were manually screened to identify any studies that were not captured in the initial search.

## Inclusion and exclusion criteria:

Inclusion criteria were defined to ensure the selection of relevant and high-quality studies. Eligible studies focused on the accuracy of CAD-CAM systems in RPD framework fabrication, provided comparisons with conventional methods, or reported quantitative data on accuracy outcomes. Both *in vitro* and *in vivo* studies published in peer-reviewed journals and available in English were included. Studies that did not focus on RPD frameworks, reviews, case reports, editorials and studies lacking sufficient data on accuracy were excluded from the review.

## Study selection:

The selection process involved two independent reviewers who initially screened the titles and abstracts of the retrieved articles to assess their relevance. Full-text articles of studies deemed potentially eligible were then reviewed for inclusion based on the predefined criteria. Any discrepancies between the reviewers were resolved through discussion, with the involvement of a third reviewer when necessary.

## Data extraction:

Data extraction was conducted independently by the two reviewers using a standardized data extraction form. Extracted data included study characteristics (*e.g.*, authors, publication year and study design), specifics of the CAD-CAM systems and conventional methods employed, accuracy measurement techniques and key outcomes related to accuracy. The primary outcome of interest was the accuracy of the RPD frameworks, as measured by parameters such as fit, marginal adaptation and dimensional stability.

## Results:

## Study Characteristics:

**Figure 1:**
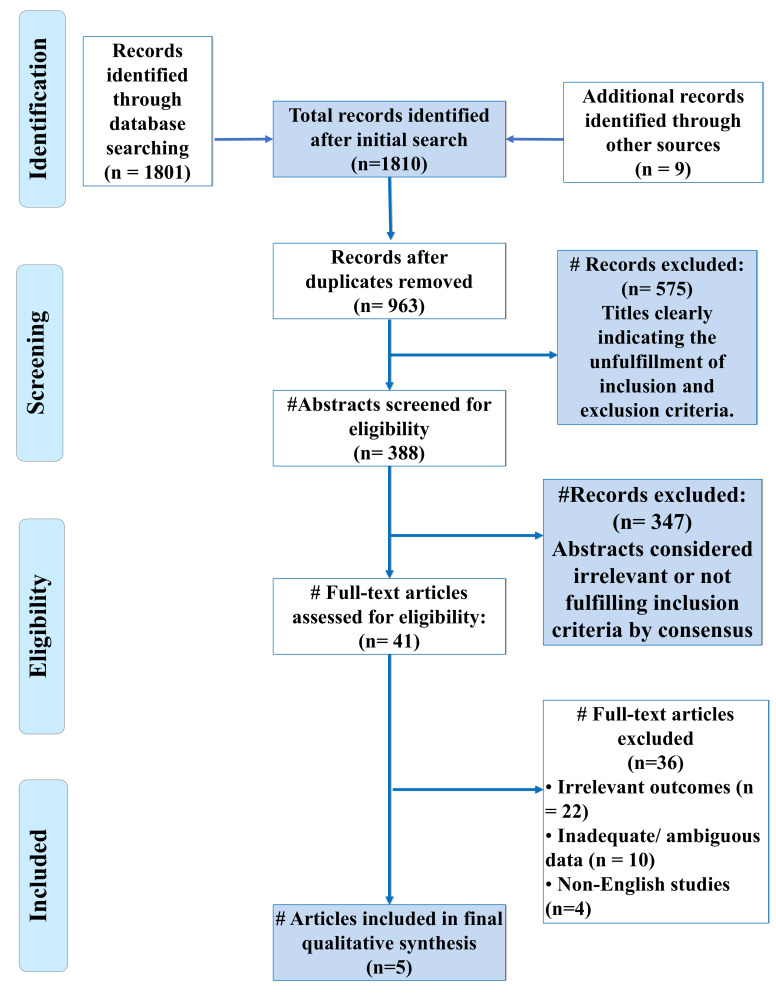
PRISMA Flowchart for the search strategy

We included a total of five in-vitro studies and one single-method study in this systematic review (Figure 1). All the studies were conducted in 2023 and the data extracted is summarized in Table 1 [13, 14, 15, 16-17]. All five studies defined different outcome measures. The studies reported of similar outcomes showing that the CAD-CAM system provided with better attachments as compared to the conventional methods. We couldn't fetch the Confidence intervals (CI) for two studies. The other three studies had details of the measurements mentioned.

## Data synthesis:

Data synthesis involved a qualitative analysis of the included studies. The results were discussed in relation to the strengths and limitations of CAD-CAM systems in RPD framework fabrication, with considerations for their clinical implications and recommendations for future research.

## Quality assessment:

The quality of the included studies was evaluated using a modified version of the Cochrane risk of bias tool for *in vitro* studies and the Newcastle-Ottawa Scale for *in vivo* studies. The assessment focused on identifying potential biases such as selection bias, performance bias, detection bias and reporting bias. The overall quality of the evidence was graded using the Grading of Recommendations, Assessment, Development and Evaluation (GRADE) approach [12].

The Database of Abstract of Reviews of Effects (DARE) tool (Table 2). The studies have the highest risk of bias in the details of the included studies. The overall quality of the articles was good. Only two studies reported partial bias.

## Discussion:

Two examples of digital fabrication techniques that have been used recently to produce RPDs are CAD/CAM and RP systems. Better functional and cosmetic results, faster fabrication times, precise design of the component pieces of the denture frame and improved fit and quality in RPD frameworks are only a few advantages of digital technology [18]. Thus, the aim of this systematic investigation was to compare and assess the fit accuracy of RPD assemblies and frameworks that were created conventionally versus digitally. Clinical trials conducted both *in vivo* and *in vitro* were incorporated to obtain meaningful data for this investigation [19]. Several studies yielded positive findings when evaluating the fit of RPDs created utilizing RP techniques [6, 7- 8]. However, RPDs manufactured using RP techniques showed appreciable anomalies in their fitting, while RPDs made using a milling approach had a considerably better framework fit than the traditional ones, according to an *in vitro* experiment by Arnold *et al.* [20]. In the majority of trials, RPDs made using the digital technique showed better fit accuracy [6, 7- 8]. No study, however, looked at the long-term clinical performance. Furthermore, a variety of methods have been reported in the literature to evaluate the accuracy and fit of RPD frameworks, including visual inspection, pressing tests, color mapping and indirect measurements of the gap filled with an imprint material [13, 14, 15, 16- 17]. Soltanzadeh *et al.* [21] found that the 3D-printed frameworks lacked the fit precision of the traditionally made RPD frameworks; color mapping was conducted utilizing sophisticated metrology software as an assessment tool. Chen *et al.* [22] also showed that standard RPD frameworks outperformed them in long span partly edentulous arches. The analyzed five investigations found that the digitally constructed RPD frameworks were more accurate than the conventional ones. The experiments included a variety of assessment and construction techniques.

SLM was utilized by Alexandrino *et al.* [23] to construct the Co-Cr alloy framework. The evaluation was completed by five doctors and entailed rating a survey containing seven framework-related elements. The findings indicated that the single digital production method was the most effective. Almufleh *et al.* [24] looked at how satisfied patients were with RPDs created with both conventional and laser-sintering processes. The prosthesis created with the SLS technique was found to provide more satisfaction. They stated that SLS-based RPD was more retentive, comfortable, efficient and stable and that it improved their speech and mastication. This significant difference may be related to the better mechanical properties of laser-sintered alloys, which are harder, denser and have demonstrated a better microstructural organization with higher yield strength and ultimate tensile strength than cast cobalt chromium alloys. In a separate clinical study, Mubaraki *et al.* evaluated the preservation of both conventionally and digitally processed RPDs [25]. The findings showed a correlation between a lower level of human interaction and the more retentive character of the digitally processed RPDs. Ahmed *et al.* [7] evaluated the clinical and cytological characteristics of RPDs created utilizing the SLS additive prototyping technique and found that the devices were precise, adaptable and had a good oral environment. According to cytological investigation, there were no microscopic inflammatory cells present in the normally desquamated oral epithelial cells. The results of this systematic review supported the null hypothesis, which stated that the internal discrepancy of Co-Cr frameworks created by the indirect technique was similar to that of the conventional technique. Soltanzadeh *et al.* noted that structures created by traditional casting had better precise adjustment than AM groups [21]. The disparity noted in these results is probably due to differences in the study design, which includes things like sample size, assessment strategies and measuring methodologies used. Of all the CAD-CAM methods that were looked at, the direct subtractive method appeared to produce the best results. This procedure reduces the need for adjustments and facilitates polishing due to its improved surface finish. Similarly, Soltanzadeh *et al.* observed a worse overall adaptation of the structures generated with AM and insufficient correction of the anterior palatal strap [21]. They surmised that this discovery was probably the consequence of errors made either during the digitization or during the software's processing of the stereolithography, even though acceptable adaptation was still recognized. Arnold *et al.* found no statistically significant difference between milled modified clasps manufactured indirectly and directly [22]. The researchers discovered that the direct AM group showed worse vertical adjustment (P<.05) than the indirect group.

## Conclusion:

CAD-CAM systems produce removable partial denture frameworks with superior fit, trueness and dimensional accuracy compared to conventional methods, as evidenced by improved retention forces, better marginal adaptation and fewer fabrication errors. Despite these advantages, limitations such as variations in outcomes between milling and additive manufacturing techniques, high costs and the need for skilled operators persist. Further research is needed to validate the long-term clinical performance of CAD-CAM fabricated frameworks and to address gaps in standardization across digital workflows.

## Figures and Tables

**Table 1:** Data extracted from the studies included in the present systematic review

**Author name**	**Year**	**Title**	**Type of study**	**Parameter compared**	**CAD-CAM**	**Conventional**	**Outcome**
Selim *et al.*	2023 [13]	Evaluation of the retention of conventional versus CAD/CAM fabricated extra coronal attachments for removable partial denture (*in vitro* study)	*In-vitro*	Mean force needed to dislodge the female clip before and after cyclic loading	46.69N and 32.13N	59.07N and 42.44N	CAD/CAM extra-coronal attachment: significantly higher retentive force than conventional.
Feng *et al.*	2023 [14]	A method to improve positioning of denture teeth on denture bases for CAD-CAM complete dentures: A dental technique	Single method	accurate seating and bonding			CNC-milled CDs: better fit, enhanced mechanical properties, biocompatible, higher patient satisfaction.
Ishioka *et al.*	2023 [15]	Morphological Comparison of Residual Ridge in Impression for Removable Partial Denture between Digital and Conventional Techniques: A Preliminary *In-vivo* Study	In-vitro	Vertical and horizontal displacements (VD and HD)	184.4 (291.1)	93.8 (194.1)	Digital impression: greater displacement in residual ridge height than conventional.
Grymak *et al.*	2023 [16]	Effect of various printing parameters on the accuracy (trueness andprecision) of 3D-printed partial denture framework	*In-vivo*	Trueness of the printing			3D printing materials: clinically acceptable RMSE with build angle 45°, specific layer thicknesses, highest discrepancies in posterior clasps.
Curinga MRS *et al.*	2023 [17]	Accuracy of models of partially edentulous arches obtained by three-dimensional printing: An *in vitro* study	*In-vivo*	linear measurements	5.80 (5.06-7.55)	5.82 (5.23-7.69)	Print spacing: middle build plate results in fewer printing failures.

**Table 2:** Quality assessment of the included studies using Database of Abstract of Reviews of Effects (DARE) tool

**Author Name**	**Reporting of Inclusion and Exclusion Criteria**	**Adequate Search**	**Quality assessment done for each study**	**Details of included studies noted**	**Included studies synthesized**
Selim *et al.*[13]	Yes	Yes	Yes	Yes	Yes
Feng *et al.*[14]	Yes	Yes	Yes	Partial	Yes
Ishioka *et al.*[15]	Yes	Yes	Yes	Yes	Yes
Grymak *et al.*[16]	Yes	Yes	Yes	Partial	Yes
Curinga MRS *et al.* [17].	Yes	Yes	Yes	Yes	Yes
